# MIND Diet and Hippocampal Sclerosis Among Community-Based Older Adults

**DOI:** 10.1001/jamanetworkopen.2025.26089

**Published:** 2025-08-07

**Authors:** Puja Agarwal, Sonal Agrawal, Maude Wagner, Laurel J. Cherian, Neelum T. Aggarwal, Bryan D. James, Thomas M. Holland, David A. Bennett, Lisa L. Barnes, Sue E. Leurgans, Julie A. Schneider

**Affiliations:** 1 Rush Alzheimer Disease Center, Department of Internal Medicine, Rush University Medical Center, Chicago, Illinois; 2 Department of Internal Medicine, Department of Clinical Nutrition, Rush University Medical Center, Chicago, Illinois; 3 Rush Alzheimer Disease Center, Department of Pathology, Rush University Medical Center, Chicago, Illinois; 4 Rush Alzheimer Disease Center, Department of Neurological Sciences, Rush University Medical Center, Chicago, Illinois; 5 Department of Neurological Sciences, Rush University Medical Center, Chicago, Illinois; 6 Rush Alzheimer Disease Center, Department of Psychiatry and Behavioral Sciences, Rush University Medical Center, Chicago, Illinois

## Abstract

**Importance:**

Hippocampal sclerosis (HS), defined as severe neuronal loss and astrogliosis in coronal sections of the midhippocampus cornu ammonis 1 or subiculum, is an important pathology associated with limbic-predominant age-related transactive response DNA-binding protein 43 encephalopathy neuropathological change (LATE-NC), Alzheimer disease, and dementia. The association of diet with HS or HS with LATE-NC in humans remains underexplored.

**Objective:**

To investigate the association of the Mediterranean–Dietary Approaches to Stop Hypertension Intervention for Neurodegenerative Delay (MIND) diet with HS and HS with LATE-NC.

**Design, Setting, and Participants:**

This cohort study included autopsied participants from the ongoing Rush Memory and Aging Project cohort study. Participants with dietary and neuropathological data from 2004 to 2024 were included. Data were analyzed from April 3, 2024, to May, 13, 2025.

**Exposures:**

Mean MIND diet scores (range, 0-15; higher score indicates better diet), computed from all validated food frequency questionnaires administered annually for up to 18 years before death.

**Main Outcomes and Measures:**

The outcomes of interest were presence of HS, assessed using hematoxylin and eosin staining, and presence of LATE-NC, detected by TDP-43 immunohistochemistry in 8 brain regions. For a subset of 300 participants, hippocampal neuronal loss severity was scored using a semiquantitative scale from 0, indicating none, to 5, severe, and categorized as none to mild, moderate, and severe neuronal loss.

**Results:**

Among 809 participants (mean [SD] age at death, 91.3 [6.1] years; 538 [72%] female; mean [SD] follow-up, 7.2 [4.4] years), HS was present in 82 (10.1%) participants; 71 participants (9%) had both HS and LATE-NC, and in the scored subset, 43 participants (14%) had moderate and 35 participants (12%) had severe hippocampal neuronal loss. Higher MIND diet scores were associated with lower odds of HS (odds ratio [OR], 0.78; 95% CI, 0.65 to 0.95) and HS with LATE-NC (OR, 0.79; 95% CI, 0.64 to 0.97) after controlling for age at death, sex, education, total calories, *APOE-ε4* status, AD, and vascular pathologies. In mediation analyses, the MIND diet was associated with less dementia at the time of death (β = −0.26; 95% CI, −0.36 to −0.15; *P* < .001), with an indirect association of 21% through HS (β = −0.05; 95% CI, −0.10 to −0.01; *P* = .02). A higher MIND diet score was associated with less hippocampal neuronal loss (*P* for trend = .01).

**Conclusions and Relevance:**

This cohort study of autopsied participants found that MIND diet adherence during follow-up was associated with a lower likelihood of HS, HS with LATE-NC, and hippocampal neuronal loss. The association of diet with dementia was partially mediated by its association with HS. These findings suggest that the MIND diet may reduce adverse brain health outcomes.

## Key Points

**Question:**

Is Mediterranean–Dietary Approaches to Stop Hypertension (DASH) Intervention for Neurodegenerative Delay (MIND) diet associated with hippocampal sclerosis (HS) and limbic-predominant age-related transactive response DNA-binding protein 43 encephalopathy neuropathological change (LATE-NC)?

**Findings:**

This cohort study of 809 autopsied participants found that MIND diet adherence was associated with lower odds of having HS, HS with LATE-NC, and hippocampal neuronal loss. The association between the MIND diet and HS partially mediated the association of MIND diet and cognition among autopsied participants.

**Meaning:**

These findings suggest that consuming a healthy diet, such as the MIND diet, may decrease the likelihood of HS and may support hippocampal health.

## Teaser

This cohort study examines the association of adherence to the Mediterranean–Dietary Approaches to Stop Hypertension Intervention for Neurodegenerative Delay (MIND) diet with hippocampal sclerosis among autopsied participants from a community-based cohort.

## Introduction

Hippocampal sclerosis (HS) is a common degenerative pathology of aging characterized by severe neuronal loss and astrogliosis in the cornu ammonis (CA) 1 and subiculum region of the hippocampus.^1^ HS is present in 20% of individuals with cognitive impairment^2^ and increasingly recognized as an important pathology associated with cognitive impairment and dementia beyond Alzheimer disease (AD) pathology.^3,4^ HS is also associated with limbic-predominant age-related transactive response DNA-binding protein 43 (TDP-43) encephalopathy neuropathologic changes (LATE-NC),^5^ AD pathology,^3^ and hippocampal volume.^6^ Previously, we observed that decedents with HS showed impairment in hippocampus-dependent cognitive abilities, like memory, and those with both HS and AD pathology had lower episodic memory scores than those with AD pathology alone.^7^ The fact that HS combined with other pathologies, like LATE-NC or AD, is associated with cognitive impairment or probable dementia suggests that prevention of these pathologies could result in lower risks of dementia. Thus, understanding whether the modifiable risk factors may prevent such pathology that impacts brain health is important for healthy aging.

Previous studies found diet to be a potentially modifiable risk factor associated with dementia and other age-related cognitive outcomes. In the Mediterranean–Dietary Approaches to Stop Hypertension Intervention for Neurodegenerative Delay (MIND) diet trial, both the MIND diet and a low-calorie diet were associated with improved cognition after 3 years.^8^ In observational studies, the MIND diet has been associated with slower cognitive decline,^9,10^ reduced Alzheimer dementia risk,^11^ better motor function,^12^ greater brain volume,^13^ higher cognitive resilience,^14^ and less AD pathology.^8^ However, it is unknown whether the MIND diet is associated with other dementia-related pathologies, including HS, LATE-NC (prevalent in individuals with HS), and hippocampal neuronal loss. Therefore, this study investigated the association of the MIND diet with HS, HS with LATE-NC, and hippocampal neuronal loss using postmortem brain tissue from a community-based sample of older adults. We further investigated whether the association of the MIND diet with a dementia diagnosis proximate to death was mediated by HS.

## Methods

### Study Participants

**Figure 1.**
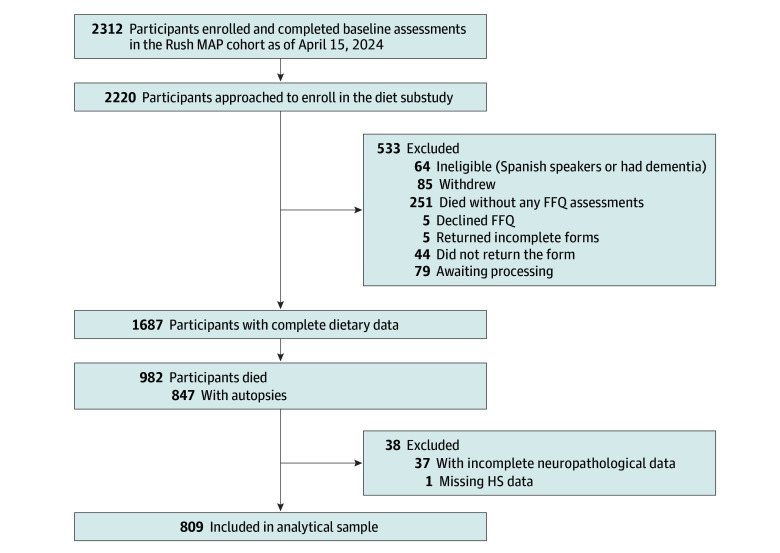
Selection of Analytical Group From the Ongoing Rush Memory and Aging Project (MAP) Cohort FFQ indicates food frequency questionnaire; HS, hippocampal sclerosis.

This cohort study was approved by the institutional review board of Rush University. The study involved autopsied participants from the Rush Memory and Aging Project (MAP). MAP, initiated in 1997, is an ongoing clinical-pathologic longitudinal cohort of older adults enrolled without known dementia and followed-up annually until death.^15^ All MAP participants sign an informed consent for annual assessments and an Anatomic Gift Act for brain donation at the time of death. As of April 15, 2024, 2312 participants enrolled and completed baseline assessments in the MAP cohort. Our analytical group includes 809 MAP decedents (Figure 1). This study is reported following the Strengthening the Reporting of Observational Studies in Epidemiology (STROBE) reporting guideline.

### Dietary Assessment

During the follow-up before death, study participants underwent annual dietary assessments using a validated food frequency questionnaire (FFQ).^16,17^ The 144-item FFQ used in MAP from 2004 to 2016 is a modified version of the Harvard FFQ. As food options and availability have changed and nutrition knowledge for brain health outcomes has evolved, the FFQ was updated in 2017 to include more foods (eg, types of berries, green leafy vegetables, whole grains). To avoid increasing participant burden, when new food items were added, some items consumed most rarely by 95% of participants were dropped (eg, frappé, chocolate milk, taco, eggroll), resulting in a 142-item FFQ with an open-ended question on other common foods consumed at least once per week. Total calories and nutrient levels were computed at the Rush Alzheimer Disease Center using methods previously described.^9^ The updated FFQ was externally validated using the MIND trial baseline data, where correlations between dietary nutrients obtained from the FFQ and plasma nutrient levels were substantial (*r* = 0.20-0.46; *P* < .001) (eTable 1 in Supplement 1).^18^ The FFQ captures usual dietary intake in the past year and is validated in older adults, irrespective of their cognitive abilities^16^ as recall of usual food items consumed is not dependent on immediate recall or episodic memory.^19^ FFQ is an established method to capture diet patterns for older adults in large cohorts^20^ and potentially invalid forms were removed during quality checks (ie, <700 or >4000 calories for men; <500 or >3800 calories for women). Of 7622 FFQs processed at the time of these analyses, only 76 (1%) were removed.

The MIND diet score is the score for 15 dietary components (range: 0-15; higher score indicates a better diet).^9^ This includes adequate or recommended consumption of 10 brain-healthy food groups (green leafy vegetables, other vegetables, nuts, berries, beans, whole grains, fish, poultry, olive oil, and wine) and avoiding or limiting 5 unhealthy food groups (red meat, butter or stick margarine, full-fat cheese, pastries and sweets, and fried and fast food)^9^ where each food group contributed a point (eTable 2 in Supplement 1). In this study, we aligned our lists of healthy foods with the current literature, the FFQ, and the intervention used for the MIND trial,^21^ with updates from original MIND diet scoring^9^ (eTable 2 in Supplement 1). We found no evidence of accelerated dietary changes approaching death (eFigure 1 in Supplement 1). We used the mean MIND diet scores for each participant calculated from repeated (nearly 4) FFQs assessed during follow-up.

### Brain Neuropathology Outcomes

Brain autopsy was performed postmortem and sectioned as per the standardized protocol to preserve the tissues. Pathological evaluations methods for the study are described in previous publications.^22^

#### Hippocampal Sclerosis

**Figure 2.**
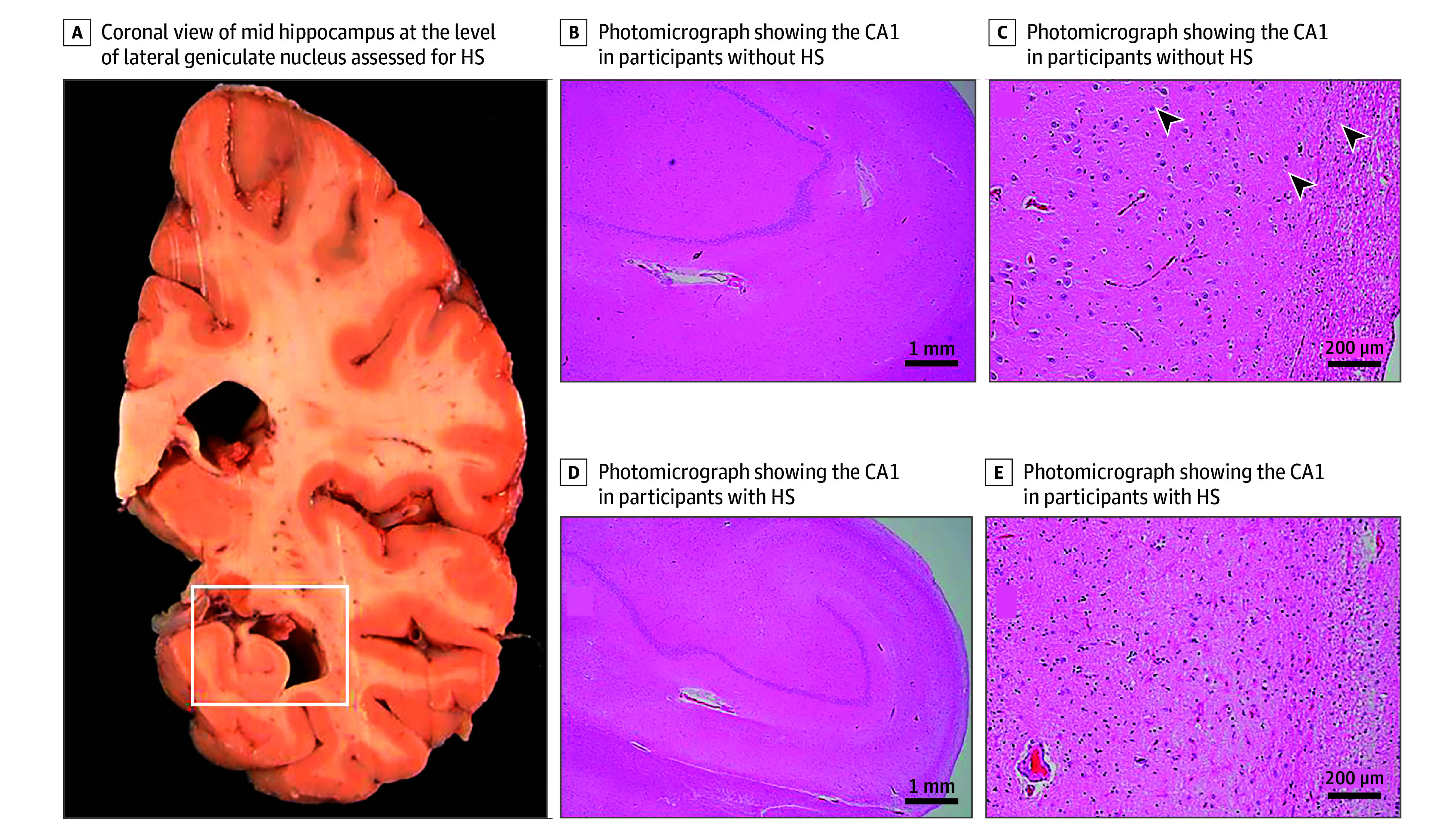
Hippocampal Sclerosis (HS) Pathology Higher magnification photographs (C and E) show the pyramidal neurons in the CA1 in a participant with no sclerosis (C, arrows) and astrogliosis with no neurons in a participant with HS (E).

The presence of HS (based on hematoxylin and eosin staining) was determined based on the severity of neuronal loss and gliosis in midhippocampus CA1 and/or subiculum. This evaluation was done unilaterally in a coronal section of the midhippocampus at the level of the lateral geniculate body. We deemed HS present if at least 1 sector has severe neuronal loss and gliosis (Figure 2). Furthermore, we also defined hippocampal neuronal loss for a subsample of 300 participants. The severity was graded from 0 to 5, where 0 indicates none or minimal neuronal loss (0%-5%); 1, mild (6%-25%); 2, mild to moderate (26%-50%); 3, moderate (51%-75%); 4, moderate to severe (76%-90%); and 5, severe (>90%).^23^ In this study, hippocampal neuronal loss was classified into 3 levels by combining the grades, with 1 indicating none, mild, or mild to moderate; 2, moderate or moderate to severe; and 3, severe.

#### LATE-NC

TDP-43 immunohistochemistry was performed on 8 brain regions using phosphorylated monoclonal TAR5P-1D3 TDP-43 antibody (pS409/410; 1:100; Ascenion). Since 2015, this antibody has been obtained from MilliporeSigma. TDP-43 cytoplasmic inclusions in neurons and glia were counted and classified into 4 stages of TDP-43 distribution according to LATE consensuses guidelines^5^: stage 0 indicates no TDP-43 inclusions; stage 1, TDP-43 present in the amygdala only; stage 2, TDP-43 present in amygdala and limbic regions; and stage 3, TDP-43 also present in neocortical regions. The presence of LATE-NC was defined as TDP-43 stages 2 and 3.

Most, but not all, incidents of HS in aging are seen in participants with LATE-NC.^24^ Thus, we also assessed HS with LATE-NC as an outcome.

### Other Covariates

Age at death was calculated from dates of birth and death. At study enrollment, participants reported sex and years of education.^25^ Polymorphic DNA Technologies performed the *APOE* genotyping (we used a dichotomous variable: those with and without ≥1 *APOE-ε4* allele).^26^ The analytical sample included primarily self-reported non-Hispanic White older adults (14 Black participants [2%]; 10 Latino participants [1%]; 783 White participants [97%], and 2 participants [<1%] identified as other race); thus, race was not considered as a covariate. Mean total calories per day were obtained from all repeated FFQs assessed during the follow-up. Final consensus Alzheimer dementia diagnosis at the time of death was based on all available clinical data reviewed by a neurologist with expertise in dementia.^27,28^ Other covariates of interest were pathologic variables (AD,^29^ amyloid-β, phosphorylated tau, moderate or severe cerebral arteriolosclerosis,^30^ and atherosclerosis^31^), body mass index (BMI),^32^ lifestyle variables (physical^33^ and social activity^34^), and vascular diseases burden^35^ (heart attack, claudication, stroke and congestive heart failure). Details on all these covariates are presented in the eMethods in Supplement 1.

### Statistical Analysis

Separate binary logistic regression models for the presence of HS, HS with LATE-NC, LATE-NC, and the severity of hippocampal neuronal loss (ordinal model in a subsample) were used. The mean of the MIND diet scores was assessed as a continuous variable and separately modeled in tertile groups (tertiles based on overall active and alive participants), with the lowest tertile as the reference category. The basic model was adjusted for age at death, sex, education, and total calorie intake. Then, we further adjusted our basic model for *APOE-ε4* status and ADNC (model A; and further on cerebral atherosclerosis and arteriolosclerosis (model B). The possibility of a nonlinear association between the MIND diet and each outcome was investigated using natural cubic splines with 2 inner knots located at the 33rd and 66th percentiles of the MIND distribution.

As secondary analyses, we considered additional potential confounders. For the HS and HS with LATE-NC outcomes, our basic model with *APOE-ε4* was additionally adjusted for hippocampal β-amyloid load and overall β-amyloid load and phosphorylated tau tangle density, and model B was further adjusted for vascular disease burden, physical activity, social activity, and BMI. For the neuronal loss outcome, the basic model with *APOE-ε4* was further adjusted for LATE-NC. Additionally, we added multiplicative terms to explore the interaction of diet with age at death (≥90 or <90 years), *APOE-ε4* status, and sex, separately. Lastly, we conducted a mediation analysis using the PROC CAUSALMED program^36^ to investigate whether the association of the MIND diet with Alzheimer dementia (final consensus at death) was mediated by HS after controlling for age at death, sex, education, and total calories. As sensitivity analyses, we removed participants with moderate to severe cognitive impairment (ie, with Mini-Mental State Exam scores^37^ <20) and FFQs assessed in last 3 years before death.

All statistical analyses were performed using SAS version 9.4 (SAS Institute). Associations were considered significant at 2-sided *P* < .05. Data were analyzed from April 3, 2024, to May, 13, 2025.

## Results

Characteristics of the 809 autopsied study participants (mean [SD] age at death, 91.2 [6.1] years; 538 [72%] female; mean [SD] follow-up, 7.2 [4.4] years) are presented in Table 1. These characteristics were similar across the tertiles of the MIND diet score, except that compared with the lowest tertile, participants in the highest tertile were more likely to be female, have more years of education, and less likely to have dementia (Table 1). Of 809 participants, 82 (10.1%) had HS and 71 (8.8%) had HS with LATE-NC. Overall, 529 participants (65%) had ADNC, 299 participants (37%) had LATE-NC (with or without HS), 209 participants (26%) had cerebral atherosclerosis, and 252 participants (31%) had arteriolosclerosis. Proximate to death, 301 participants (37%) had Alzheimer dementia. As expected, a higher MIND diet score was associated with lower odds of a dementia diagnosis proximate to death (odds ratio [OR], 0.77; 95% CI, 0.68-0.87).

**Table 1.** Characteristics of Autopsied Participants of the Memory and Aging Project With MIND Diet and Hippocampal Sclerosis Data

Characteristic	Mean (SD)			
	Overall (N = 809)	Tertile of MIND diet score		
		1 (n = 312)	2 (n = 237)	3 (n = 260)
MIND diet score^a^	7.2 (1.4)	5.7 (0.7)	7.1 (0.3)	8.6 (0.8)
Age at death, y	91.2 (6.1)	90.6 (6.6)	91.7 (5.7)	91.8 (5.9)
Age at first FFQ, y	84.1 (6.0)	84.1 (5.6)	84.1 (5.6)	83.9 (5.5)
Education, y	15.0 (2.9)	14.2 (3.0)	15.2 (2.7)	15.8 (2.7)
Sex, No. (%)				
Female	538 (72)	213 (68)	174 (73)	198 (76)
Male	224 (28)	99 (31)	63 (27)	62 (24)
*APOE-ε4* status, No. (%)^b^	160 (20)	56 (18)	57 (24)	47 (18)
Total calories during follow-up, kcal/d	1820 (517)	1812 (586)	1817 (485)	1831 (454)
Physical activity, h/wk^c^	3.0 (3.2)	2.2 (2.9)	3.2 (2.9)	3.7 (3.7)
Social activity score^d^	2.5 (0.6)	2.4 (0.6)	2.5 (0.5)	2.7 (0.6)
BMI^e^	26.7 (4.8)	27.1 (4.9)	26.7 (5.3)	26.2 (4.3)
Vascular disease burden^f^	0.5 (0.7)	0.5 (0.8)	0.5 (0.7)	0.4 (0.7)
Alzheimer dementia diagnosis, No. (%)^g^	301 (37)	135 (43)	91 (38)	75 (29)
ADNC, No. (%)^h^	529 (65)	194 (62)	161 (68)	174 (67)
Cerebral atherosclerosis, No. (%)^i^	209 (26)	88 (28)	52 (22)	69 (26)
Cerebral arteriolosclerosis, No. (%)^c^	252 (31)	91 (29)	64 (27)	97 (37)
LATE-NC, No. (%)^j^	299 (37)	108 (35)	93 (39)	98 (38)

Abbreviations: ADNC, Alzheimer’s Disease Neuropathologic Change; BMI, body mass index (calculated as weight in kilograms divided by height in meters squared); FFQ, food frequency questionnaire; LATE-NC, limbic-predominant age-related transactive response DNA-binding protein 43 encephalopathy-neuropathological change; MIND, Mediterranean–Dietary Approaches to Stop Hypertension Intervention for Neurodegenerative Delay.

^a^ Mean intake during the duration of follow-up.

^b^ Missing data on 59 participants.

^c^ Missing data on 2 participants.

^d^ Missing data on 3 participants.

^e^ Missing data on 39 participants.

^f^ Missing data on 2 participants. Vascular disease burden was calculated as self-reported presence of any 1 of these vascular diseases: claudication, stroke, heart failure and myocardial infarction (range: 0-4); assessed at first dietary assessment.

^g^ Final consensus Alzheimer dementia diagnosis. Missing data on 7 participants.

^h^ High or intermediate likelihood based on National Institute on Aging-Alzheimer Association criteria for the pathologic diagnosis of Alzheimer disease.

^i^ Missing data on 1 participant.

^j^ Presence of limbic-predominant age-related TDP-43 encephalopathy-neuropathological change. Missing data on 9 participants.

### MIND Diet, Hippocampal Sclerosis, and LATE-NC

**Figure 3.**
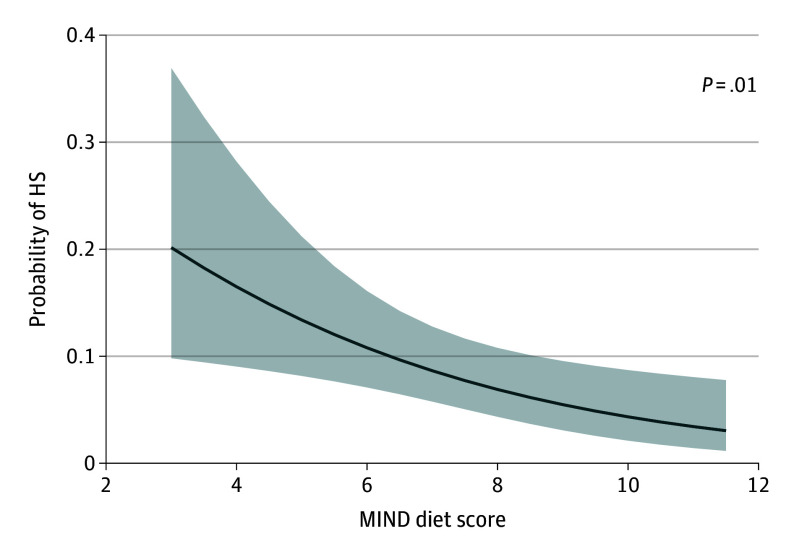
Association of Mediterranean–Dietary Approaches to Stop Hypertension (DASH) Intervention for Neurodegenerative Delay (MIND) Diet Score and Risk of Hippocampal Sclerosis (HS) Model controlled for age at death, sex, education, *APOE-ε4* status, calories, Alzheimer disease, and vascular pathologies.

A higher MIND diet score was associated with lower odds of having HS (OR, 0.79; 95% CI, 0.66-0.95) in models controlled for age at death, sex, education, and calories. This association was retained when additionally controlled for *APOE-ε4* status and ADNC (model A) and further for other vascular pathologies (model B) (Table 2). Each additional point in MIND diet score was associated with 22% lower odds of having HS (Figure 3). In secondary analysis where we controlled for hippocampal β-amyloid load (OR, 0.75; 95% CI, 0.62-0.92) or overall β-amyloid load and phosphorylated tau-tangle density (OR, 0.76; 95% CI, 0.63-0.93) instead of ADNC, the results did not change. After adjusting model B for BMI, physical and social activity, and vascular disease burden, the associations were retained (eTable 3 in Supplement 1). We saw no association between MIND diet score and LATE-NC in the basic (OR, 1.00; 95% CI, 0.91-1.11) or further adjusted models. Overall, a higher MIND diet score was also associated with lower odds of having HS with LATE-NC in the basic model (OR, 0.80; 95% CI, 0.66-0.98) and models adjusted for ADNC and vascular pathology (Table 2). There was no evidence of nonlinear associations (eFigure 2 in Supplement 1).

**Table 2.** Association of the MIND Diet With HS, HS With LATE-NC, and Hippocampal Neuronal Loss Severity

Outcome	Model A^a^		Model B^b^	
	OR (95% CI)	*P* value for trend	OR (95% CI)	*P* value for trend
**Hippocampal sclerosis**				
No.	750	NA	747	NA
MIND diet score, per 1-point increase	0.79 (0.65-0.95)	NA	0.78 (0.65-0.95)	NA
MIND diet score tertile				
1^c^	1 [Reference]	.03	1 [Reference]	.03
2^d^	0.56 (0.31-0.99)		0.56 (0.31-1.02)	
3^e^	0.53 (0.30-0.95)		0.53 (0.30-0.97)	
**Hippocampal sclerosis with LATE-NC**				
No.	741	NA	739	NA
MIND diet score, per 1-point increase	0.80 (0.65-0.97)	NA	0.79 (0.64-0.97)	NA
MIND diet score tertile				
1^c^	1 [Reference]	.049	1 [Reference]	.04
2^d^	0.63 (0.34-1.17)		0.64 (0.35-1.20)	
3^e^	0.53 (0.28-1.00)		0.52 (0.28-0.98)	
**Hippocampal neuronal loss in subsample** ^f^				
No.	249	NA	248	NA
MIND diet score, per 1-point increase	0.81 (0.65-1.01)	NA	0.86 (0.62-1.07)	NA
MIND diet score tertile				
1^c^	1 [Reference]	.01	1 [Reference]	.04
2^d^	0.47 (0.22-0.99)		0.55 (0.48-1.11)	
3^e^	0.39 (0.19-0.79)		0.45 (0.21-0.93)	

Abbreviations: HS, hippocampal sclerosis; LATE-NC, limbic-predominant age-related transactive response DNA-binding protein 43 encephalopathy-neuropathological change; MIND, Mediterranean–Dietary Approaches to Stop Hypertension Intervention for Neurodegenerative Delay; NA, not applicable; OR, odds ratio.

^a^ Controlled for age at death, sex, education, calories, *APOE-ε4* status, and Alzheimer’s Disease Neuropathologic Change, ie, basic model + *APOE-ε4* status and ADNC.

^b^ Controlled for Model A + cerebral atherosclerosis and arteriolosclerosis.

^c^ Median (IQR) score, 6.0 (5.5-6.3).

^d^ Median (IQR) score, 7.0 (6.9-7.5).

^e^ Median (IQR) score, 8.4 (8.0-9.0).

^f^ Ordinal logistic models to assess severity of neuronal loss (ordinal variable) on those with hippocampal neuronal loss data.

### MIND Diet and Hippocampal Neuronal Loss

Given that HS is a severe form of neuronal loss, to understand the association of diet with the spectrum of neuronal loss, we assessed a subsample of 300 participants with hippocampal neuronal loss data (mean [SD] loss category, 1.9 [1.1]), including 43 participants (14%) with moderate and 35 participants (12%) with severe hippocampal neuronal loss. In the basic model, controlling for demographics and calorie intake, the highest tertile of the MIND diet, compared with the lowest tertile, had 48% lower odds of sever neuronal loss (OR, 0.52; 95% CI, 0.27-0.98). Highest MIND diet tertile was associated with less hippocampal neuronal loss when further adjusted for *APOE-ε4* status, ADNC, and other vascular pathologies (Table 2). In secondary analysis controlling for *APOE-ε4* status and LATE-NC, continuous MIND diet score was associated with less hippocampal neuronal loss (OR, 0.75; 95% CI, 0.59-0.95).

### Interaction Models, Mediation, Sensitivity, and Other Analysis

Animal and human studies have demonstrated that *APOE-ε4* is associated with lipid metabolism, amyloid burden, and gliosis, and interacts with diet-induced metabolic impairments in female animal models^29,30,31^; however, in our analysis, there were no interactions between the MIND diet and age at death (*P* for interaction = .10), sex (*P* for interaction = .68), or *APOE-ε4* status (*P* for interaction = .39) (eTable 4 in Supplement 1). We explored whether HS mediated the association of diet with Alzheimer dementia using mediation analysis. The MIND diet was associated with Alzheimer dementia at the last clinical visit (β = −0.26; 95% CI, −0.36 to −0.15; *P* < .001), with an indirect association of 20.7% through HS (β = −0.05; 95% CI, −0.10 to −0.01; *P* = .02).

In the first sensitivity analysis, we removed 32 participants with Mini-Mental State Exam scores below 20 at their first FFQ assessment. We found similar results for the fully adjusted model (model B): MIND diet was associated with lower odds of HS (OR, 0.80; 95% CI, 0.66-0.97) and with hippocampal neuronal loss among 247 participants with neuronal loss data (highest vs lowest tertile: OR, 0.47; 95% CI, 0.23-0.99). Results were almost similar in a second sensitivity analysis removing FFQs administered within 3 years of death (eTable 5 in Supplement 1). Among similar plant-based dietary patterns, including Mediterranean and DASH diet (correlated with MIND diet) (eTable 6 in Supplement 1), we found only the Mediterranean diet was associated with HS but not neuronal loss (eTable 7 in Supplement 1).

## Discussion

In this neuropathologic cohort study of autopsied participants from a community cohort, adherence to the MIND diet was associated with lower odds of HS when controlled for demographic factors, calorie intake, *APOE-ε4* status, AD, and vascular pathologies. Overall, each 1-point increase of the MIND diet score was associated with almost 22% lower odds of HS. Additionally, the MIND diet was associated with lower odds of HS with LATE-NC. Importantly, HS partially mediated the association of the MIND diet with dementia by almost 21%. However, given the role of mixed pathologies in dementia,^38^ multiple mechanisms could explain this mediation. In a subsample, higher MIND diet adherence was associated with less hippocampal neuronal loss even after controlling for other common age-related pathologies. Overall, these findings support the role of the MIND diet for a common degenerative pathology of aging, specifically HS, and hippocampal neuronal loss in aging. These results may partially explain the association of diet with cognitive aging and the role of a healthy diet in dementia risk and other brain health outcomes.

To our knowledge, this is the first study showing association of diet with HS and hippocampal neuronal loss in humans. The pathogenesis of HS is not clear but may be neurodegenerative and/or vascular. These findings support a previously documented association of diet with slower decline in episodic memory in the same cohort,^9^ the cognitive domain primarily affected by the hippocampus and related structures in the medial temporal lobe. This also corresponds with studies reporting an association of the MIND diet with higher hippocampal volume,^39,40^ and we know HS is associated with smaller hippocampal volume.^6^ As MIND has unique dietary components eg, berries and leafy-greens, future larger studies on the contribution of individual dietary components of different diet plans are warranted. These findings suggest further exploration of the underlying mechanisms between diet and neuroimaging, neuropathologic, and memory outcomes is needed.

These results are also consistent with animal studies on diet and neurobiological mechanisms of hippocampus health. A Western diet (high in fat, sugar, and processed foods) exhibited long-lasting deficits in hippocampal-dependent memory with disrupted hippocampal acetylcholine signaling in rats.^41^ A high-fat diet reduced hippocampal neuroplasticity in mice via activated hippocampal microglia and lipid accumulation^42^ and increased inflammatory cytokines and activated microglia in the brain^43^ and overall enhanced astrogliosis,^44^ resulting in behavioral difficulties. Whereas ω3 fatty acid downregulates microglial inflammation.^45^ Healthy diets are rich in various nutrients, essential fats, and bioactive compounds with antioxidant and anti-inflammatory properties and are known to reduce systemic inflammation and oxidative stress. The MIND diet also avoids or limits proinflammatory components, like high-fat, high-sugar, and processed foods. To further understand the mechanisms linking diet and nutrients with the overall brain health, investigating diet’s relationship with other biomarkers of neuroinflammation and oxidative stress in the brain will be critical.

### Strengths and Limitations

The primary strengths of this study include a large autopsy sample of 809 community-dwelling older adults without known dementia at baseline, multiple annual dietary assessments using a comprehensive and validated FFQ for older adults, other structured annual clinical assessments, and standardized neuropathologic measures. High autopsy rates strengthen generalizability to the entire cohort. Multiple diet assessments during follow-up reduce measurement error by averaging within-person variation; however, potential reverse causation due to accumulating neuropathologic burden remains a limitation. Other limitations include inability to establish causal relationships due to observational study design that measures diet during life and its association with pathology at death. We cannot exclude the possibility of important unmeasured confounding variables, including foods that were not captured in the questionnaire. The study included primarily older non-Hispanic White volunteers; thus, we cannot generalize these results to younger adults, to groups reluctant to consider brain autopsy, or to more diverse populations.

## Conclusions

Overall, this study supports that among older adults, adhering to a healthy dietary pattern, such as the MIND diet, is associated with reduced likelihood of hippocampal degeneration of aging, as indicated by HS, HS with LATE-NC, and hippocampal neuronal loss. Our findings also support that the association of the MIND diet with hippocampal health may partially explain the association of healthy diet with reduced dementia risk. Further studies should investigate potential neurobiological mechanisms explaining these links, including neuroinflammation and brain oxidative stress, 2 proposed mechanisms for the association of diet with dementia.

## Notes

Supplement 1. **eMethods.** Description of covariates
eReferences.
**eTable 1.** Updated Food Frequency Questionnaire (FFQ) validation **eTable 2.** Updated MIND diet table with 15 dietary components, cut-offs, and a list of foods included in each component for Rush Memory and Aging Project and Minority Aging Research Study **eTable 3.** Association of MIND diet (continuous score) with hippocampal sclerosis **eTable 4.** Models to test the interaction of MIND diet with age at death, sex, and APO-*ε*4 allele for the association of MIND diet with hippocampal sclerosis **eTable 5.** Sensitivity analysis (after removing any FFQ done in last 3 years before death) for association of MIND diet with hippocampal sclerosis **eTable 6.** Spearman Correlation coefficient for MIND, Mediterranean, and DASH diet scores in our analytical sample **eTable 7.** Association of Mediterranean and DASH diet scores with hippocampal sclerosis (HS), HS with LATE, and hippocampal neuronal loss in our analytical sample **eFigure 1.** Marginal trajectory of the MIND diet score estimated in the years before death when considering a linear function of time or a nonlinear function of time **eFigure 2.** Spline curves to test the nonlinearity for the association of MIND diet score and risk of Hippocampal sclerosis (HS), HS with LATE-NC, and hippocampal neuronal loss using natural cubic splines with 2 knots located at 33th and 66th percentile of the MIND diet score distribution

Supplement 2.
Data Sharing Statement
