# Error in Figure 3

**DOI:** 10.1001/jamanetworkopen.2025.37570

**Published:** 2025-09-18

In the Original Investigation titled “MIND Diet and Hippocampal Sclerosis Among Community-Based Older Adults,”^1^ published August 7, 2025, the tick marks in the y-axis of Figure 3 were labeled incorrectly. This article has been corrected.^1^
