# Supplementary material for: MIND Diet and Hippocampal Sclerosis Among Community-Based Older Adults
Source: JAMA Netw Open. 2025 Aug 7;8(8):e2526089. doi: 10.1001/jamanetworkopen.2025.26089 (PMC12332637; doi:10.1001/jamanetworkopen.2025.26089)
Supplement: Supplement 1. — eMethods. Description of covariates eReferences. eTable 1. Updated Food Frequency Questionnaire (FFQ) validation eTable 2. Updated MIND diet table with 15 dietary components, cut-offs, and a list of foods included in each component for Rush Memory and Aging Project and Minority Aging Research Study eTable 3. Association of MIND diet (continuous score) with hippocampal sclerosis eTable 4. Models to test the interaction of MIND diet with age at death, sex, and APO-ε4 allele for the association of MIND diet with hippocampal sclerosis eTable 5. Sensitivity analysis (after removing any FFQ done in last 3 years before death) for association of MIND diet with hippocampal sclerosis eTable 6. Spearman Correlation coefficient for MIND, Mediterranean, and DASH diet scores in our analytical sample eTable 7. Association of Mediterranean and DASH diet scores with hippocampal sclerosis (HS), HS with LATE, and hippocampal neuronal loss in our analytical sample eFigure 1. Marginal trajectory of the MIND diet score estimated in the years before death when considering a linear function of time or a nonlinear function of time eFigure 2. Spline curves to test the nonlinearity for the association of MIND diet score and risk of Hippocampal sclerosis (HS), HS with LATE-NC, and hippocampal neuronal loss using natural cubic splines with 2 knots located at 33th and 66th percentile of the MIND diet score distribution [file jamanetwopen-e2526089-s001.pdf]

Supplemental Online Content

Agarwal P, Agrawal S, Wagner M, et al. MIND diet and hippocampal sclerosis among community-based older adults. *JAMA Netw Open*. 2025;8(8):e2526089. doi:10.1001/jamanetworkopen.2025.26089

eMethods. Description of covariates

eReferences.

eTable 1. Updated Food Frequency Questionnaire (FFQ) validation

eTable 2. Updated MIND diet table with 15 dietary components, cut-offs, and a list of foods included in each component for Rush Memory and Aging Project and Minority Aging Research Study

eTable 3. Association of MIND diet (continuous score) with hippocampal sclerosis

eTable 4. Models to test the interaction of MIND diet with age at death, sex, and APO-ε4 allele for the association of MIND diet with hippocampal sclerosis

eTable 5. Sensitivity analysis (after removing any FFQ done in last 3 years before death) for association of MIND diet with hippocampal sclerosis

eTable 6. Spearman Correlation coefficient for MIND, Mediterranean, and DASH diet scores in our analytical sample

eTable 7. Association of Mediterranean and DASH diet scores with hippocampal sclerosis (HS), HS with LATE, and hippocampal neuronal loss in our analytical sample

eFigure 1. Marginal trajectory of the MIND diet score estimated in the years before death when considering a linear function of time or a nonlinear function of time

eFigure 2. Spline curves to test the nonlinearity for the association of MIND diet score and risk of Hippocampal sclerosis (HS), HS with LATE-NC, and hippocampal neuronal loss using natural cubic splines with 2 knots located at 33th and 66th percentile of the MIND diet score distribution

This supplemental material has been provided by the authors to give readers additional information about their work.

© 2025 Agarwal P et al. *JAMA Network Open*.

© 2025 Agarwal P et al. *JAMA Network Open*.

Supplementary Materials:

e.Methods: Description of covariates

Pathologic variables: The National Institute on Aging-Alzheimer's Association (NIA-AA) criteria for the pathologic diagnosis of AD was used[1] i.e. a combination of neurofibrillary tangles (Braak), neuritic plaque score (CERAD), and amyloid beta plaque score (Thal). The Alzheimer's Disease neuropathologic change (ADNC) is present if intermediate or high likelihood is scored; if low or no likelihood, ADNC will be treated as not present. Amyloid-beta load and phosphorylated tau as previously described were assessed using immunohistochemistry at multiple brain regions (eight regions-entorhinal, mid-frontal, inferior temporal, angular gyrus, calcarine, anterior cingulate, superior frontal cortices, and hippocampus)[2]. A composite continuous summary measure of the total beta-amyloid load was generated using the mean percent area of each region occupied and then averaging the values for eight regions [3] and phosphorylated tau tangle density was calculated using the total number of tangles divided by area of mm^2 and then averaging the values for eight regions. We also used the amyloid score count specific to the hippocampus as a covariate. All overall and regional amyloid and tangle variables were square root transformed for statistical procedures. Large vessel cerebral atherosclerosis was done by visual inspection of the circle of Willis for the involvement of each artery and the number of arteries involved scored as 0= no atherosclerosis to 6= severe atherosclerosis[4]. Cerebral arteriolosclerosis was described as histological changes found in arterioles. This was graded and coded as 0=none, 1=mild, 2=moderate and 3=severe[5].

Lifestyle variables: Body mass index (BMI), physical activity, social activity, and vascular disease conditions were collected at each annual clinical visit; in our context, we focused on each participant's report at first FFQ. BMI was computed from measured weight and self-reported height. Physical activity (hours per week based on self-reported minutes spent for walking, exercise, yard work, calisthenics, biking, and water exercise) [6],. Social activities included frequency of **late-life social activities** using 6 items which ask

© 2025 Agarwal P et al. *JAMA Network Open*.

how often during the past year participants engaged in common types of activities involving social interaction e.g. going to restaurants, sports events, bingo nights, day or overnight trips, or do unpaid community volunteer work, visit friends' or relatives' house, participate in groups or attend church. Participants are asked to rate the items on a 5-point scale, with higher values indicating more frequent participation. Item scores are averaged to yield the composite measure, with higher scores indicating greater social activity[7].

Vascular diseases: Vascular diseases burden was self-reported and included the sum of questions for the following 4 items: congestive heart failure, claudication, stroke and heart conditions (e.g., self-reported heart attack or coronary, coronary thrombosis, coronary occlusion, myocardial infarction). Each item is given a value of 0 or 1 (see response options below). The cumulative score for vascular disease burden ranges from 0 to 4 and is the total number of items reported. Higher scores indicate greater vascular disease burden. In addition to self-report, evaluation of stroke is also based on neurological exam (when available), cognitive testing, and interview of participant, with which the physician may render a diagnosis of stroke[8].

References:

1. Hyman BT, Phelps CH, Beach TG, et al. National Institute on Aging-Alzheimer's Association guidelines for the neuropathologic assessment of Alzheimer's disease. Alzheimers Dement 2012;8:1-13.
2. Boyle PA, Yu L, Leurgans SE, et al. Attributable risk of Alzheimer's dementia attributed to age-related neuropathologies. Ann Neurol 2019;85:114-124.
3. Bennett DA, Schneider JA, Wilson RS, Bienias JL, Arnold SE. Neurofibrillary tangles mediate the association of amyloid load with clinical Alzheimer disease and level of cognitive function. Archives of neurology 2004;61:378-384.
4. Arvanitakis Z, Capuano AW, Leurgans SE, Buchman AS, Bennett DA, Schneider JA. The Relationship of Cerebral Vessel Pathology to Brain Microinfarcts. Brain Pathol 2017;27:77-85.
5. Buchman AS, Leurgans SE, Nag S, Bennett DA, Schneider JA. Cerebrovascular disease pathology and parkinsonian signs in old age. Stroke 2011;42:3183-3189.

© 2025 Agarwal P et al. *JAMA Network Open*.

6. McPhillips JB, Pellettera KM, Barrett-Connor E, Wingard DL, Criqui MH. Exercise patterns in a population of older adults. American journal of preventive medicine 1989;5:65-72.
7. Buchman AS, Boyle PA, Wilson RS, Fleischman DA, Leurgans S, Bennett DA. Association between late-life social activity and motor decline in older adults. Archives of internal medicine 2009;169:1139-1146.
8. Boyle PA, Buchman AS, Wilson RS, Leurgans SE, Bennett DA. Association of muscle strength with the risk of Alzheimer disease and the rate of cognitive decline in community-dwelling older persons. Archives of neurology 2009;66:1339-1344.

Supplementary Tables

e.Table 1: Updated Food Frequency Questionnaire (FFQ) validation: Pearson correlation* and partial correlations (adjusted for various confounding factors [a,b]) between calorie-adjusted nutrients obtained from FFQ and circulating nutrient levels. Correlation between dietary nutrients obtained using the updated FFQ and plasma nutrients from the same visit among the participants of the MIND trial study (the MIND diet trial is registered at clinicaltrials.gov (NCT02817074); baseline assessments, mean age= 70.4 ± 4.2; 65 % female (Barnes LL, Dhana K, Liu X, et al. Trial of the MIND Diet for Prevention of Cognitive Decline in Older Persons. N Engl J Med 2023;389:602-611). These correlations indicate similar correlations between nutrients from the updated FFQ and plasma as published in the original FFQ validation paper and published abstract ((Tangney, C.C., Agarwal, P., Ventrelle, J., Aggarwal, N.T., Dhana, K., Arfanakis, K. and Barnes, L.L. (2023), Relative Validity of the revised RUSH Food Frequency Questionnaire and MIND diet screener: Capturing the Mediterranean-DASH Intervention for Neurodegenerative Delay (MIND) dietary pattern. Alzheimer's Dement., 19: e074843. https://doi.org/10.1002/alz.074843)). These correlations validate the use of a modified FFQ and updated processing of FFQ at Rush Alzheimer’s Disease Center to compute average nutrient intake and other dietary factors among older participants enrolled in our studies. Details on correlations presented in the table below.

© 2025 Agarwal P et al. *JAMA Network Open*.

	Dietary alpha-carotene (mcg/day)	Dietary beta-carotene (mcg/day)	Dietary Vitamin E (mg/day)	Dietary Folate (mcg/day)	Dietary Lutein (mcg/ day)	Dietary Vitamin B12 (mcg/day)	Dietary beta-cryptoxanthin (mcg/day)
Plasma alpha-carotene (mcg/l)	0.31; p<0.000* 0.29; p<0.000[a] N=440						
Plasma beta-carotene (mcg/l)		0.31; p<0.001* 0.28; p<0.001[a] N=440					
Plasma alpha-tocopherol (mcg/l)			0.17; p<0.001* 0.21; p<0.001[b] N=441				
Serum Folate (ng/ ml)				0.33* p<0.001 N=583			
Plasma Lutein (mcg/l)					0.37* p<0.001 N=441		
Plasma Vitamin B12 (pg/ml)						0.22* p<0.001 N=581	
Plasma beta-cryptoxanthin (mcg/l)							0.46 p<0.001 N=440

* Pearson correlation; [a] Partial correlation adjusted for age, smoking status, and alcohol use; [b] Partial correlation adjusted for age, and serum total cholesterol.

© 2025 Agarwal P et al. *JAMA Network Open*.

e.Table 2.a.: Updated MIND diet table with 15 dietary components, cut-offs, and a list of foods included in each component for Rush Memory and Aging Project and Minority Aging Research Study

	0	0.5	1
Healthy Dietary Components/ Recommended Food groups			
Green Leafy Vegetables [a]	≤ 2 servings/wk	> 2 to <7/wk	≥ 7 servings/wk
Other Vegetables [b]	< 5 servings/wk	≥ 5 - < 7/wk	≥ 7 servings/wk
Berries [c]	< 1 serving*/wk	≥1 - < 5/wk	≥ 5 servings*/wk
Nuts [d]	< 1 serving/wk	≥ 1 - < 5/wk	≥ 5 servings/wk
Fish (not fried) [e]	< 1 serving/ month or rarely	1-3 serving/ month or < 1 serving/ month	≥ 1 serving/wk
Poultry (not fried, skinless) [f]	< 1 serving/wk	≥ 1 - < 2/wk	≥ 2 servings/wk
Beans & Legumes [g]	< 1 serving/wk	≥ 1- < 3/wk	≥3 servings/wk

© 2025 Agarwal P et al. *JAMA Network Open*.

Whole Grains [h]	< 7 servings/wk	≥ 7 - < 21/wk	≥ 21 servings/wk
Olive Oil [i]	< 7 T/wk	≥7 - < 14 T/wk	≥ 14 T/wk
Wine [j]	>1 glass/d or never	1 glass/month – 6 glasses /wk	1 glass/d
Unhealthy Dietary Components/ Foods to limit or avoid			
Butter and stick margarine [k]	≥ 14 pat(tsp)/wk	> 7 - < 14/wk	≤ 7 pat(tsp)/wk
Full-fat Cheese [l]	≥ 7 servings/wk	> 2 – < 7/wk	≤ 2 serving/wk
Red and Processed Meat [m]	≥7 meals/week	≥4 to <7 meals/week	<4 meals/week
Fast and Fried Foods [n]	≥ 4 meal/wk	> 1 - < 4/wk	≤ 1 meal/wk
Pastries & Sweets [o]	≥ 7 servings/wk	≥ 5 - < 7 wk	< 5 servings/wk

© 2025 Agarwal P et al. *JAMA Network Open*.

TOTAL SCORE	15

e.Table 2. b. Different foods included in each MIND diet component score in e.Table 2.a.:

Food groups	Foods included
[a] Green leafy vegetable	Spinach, romaine or leaf lettuce, kale, collard greens, mustard, or other reported on open-ended question – turnip or beet greens, chard, arugula
[b] other vegetables	Tomato, broccoli, beet, corn, mixed vegetable, spinach, kale, peppers, yams/ sweet potato, zucchini/ squash/ eggplant, carrots, celery, lettuce, cabbage/coleslaw, potato (baked, not fried), peas or lima beans, green/red/yellow peppers, string beans, brussels sprouts, mushrooms, iceberg or head lettuce, onions
[c] Berries	Strawberries, Blueberries, raspberries & blackberries, or other berries reported on open-ended question- goji berries, acai berries; *one serving is considered as ½ cup considering the average intake by older adults in NHANES/ WWEIA data.
[d] Nuts	Mixed nuts, peanuts, peanut butter, other nuts
[e] Fish and Seafood (not fried)	Tuna, dark meat fish, light meat fish (not fried), shrimp/ scallops/ lobsters, not included fried fish sticks/cakes/ sandwich
[f] Poultry (not fried)	Chicken or turkey (roasted/baked/boiled/ grilled)
[g] Beans and Legumes	Beans/ lentils/ soybeans, or other reported on open-ended questions- e.g. hummus, chickpeas
[h] Whole grains	Dark bread, kasha/couscous/ bulgur/quinoa, oatmeal/ grits, High fiber whole grain cereals, whole grain pasta, bread, pizza, crackers, brown rice, breakfast bars, and popcorn
[i] Olive oil	Any olive oil as the primary oil used
[j] Wine	Red and white wine
Foods to Limit:	
[k] Butter, Margarine	Regular butter, Margarine if regular stick or tub
[l] Full-fat cheese	Full-fat cheese, cream cheese, cottage or ricotta cheese, cheese in cheeseburgers, pizza, and grilled cheese sandwich

© 2025 Agarwal P et al. *JAMA Network Open*.

[m] Red and processed meat	Cheeseburgers, hamburger, hot dogs/ bratwurst, salami/ bologna/ other deli meat slices, beef (steak, roast)/ lamb as the main dish, pork/ ham or chops as the main dish, meatballs/ meatloaf, breakfast meat like bacon or sausage
[n] Fast and fried foods	Fried chicken, French fries, chicken nuggets, fried fish, potato chips, Corn/ tortilla chips/Doritos or Fried food away from home
[o] Pastries and Sweets	Biscuits/rolls, pop tarts, snack cakes/ twinkies, Danish/sweet rolls/ pastry, donuts, cookies, pie, brownies, chocolate bars, other candy bars, other candies, ice creams, puddings, sugared beverages: soda, Hawaiian punch etc.

Note: Compared to old scoring matrix and foods included the updated MIND diet scoring with this above updated list of foods gave similar overall scores (7.84 ± 1.8 vs. 7.50 ± 1.8) that were highly correlated among 7,543 FFQs i.e. overall dietary data collected at Rush Alzheimer's Disease Center (rho=0.91, p< 0.001).

© 2025 Agarwal P et al. *JAMA Network Open*.

e.Table 3: Secondary analysis: Association of MIND diet (continuous score) with hippocampal sclerosis (HS). HS with Limbic-predominant age-related TDP-43 encephalopathy- neuropathological change (LATE-NC) and Hippocampal Neuronal loss severity adjusted for demographic and genetic factors, Alzheimer's Disease pathology, vascular pathology, BMI, lifestyle factors and vascular diseases.

	**OR (95% CI)**
**Hippocampal Sclerosis**	N=714
MIND diet score (continuous)	0.80 (0.65, 0.98)
**Hippocampal Sclerosis with LATE-NC**	N=706
MIND diet score (continuous)	0.80 (0.64, 0.99)
**Hippocampal Neuronal Loss in Subsample ***	N=242
MIND diet score (continuous)	0.89 (0.69, 1.13)

Logistic models adjusted for age at death, sex, education, APO-ε4 allele, calories, ADNC (Alzheimer's disease neuropathological changes), cerebral atherosclerosis, and arteriosclerosis pathology, BMI, physical activity, social activity, and vascular disease burden (congestive heart failure, claudication, stroke and heart conditions).

e.Table 4: Models to test the interaction of MIND diet with age at death, sex, and APO-ε4 allele for the association of MIND diet with hippocampal sclerosis (effect estimates and p for interaction).

© 2025 Agarwal P et al. *JAMA Network Open*.

Model	Model term	Effect estimates (SE)	**p-value**
**Model A**	MIND diet score (continuous)	-0.009 (0.179)	0.960
	Age at death	3.058 (1.486)	0.040
	MIND*Age at death	-0.353 (0.211)	0.095
**Model B**	MIND diet score (continuous)	-0.291 (0.116)	0.012
	sex	-0.7911 (1.48)	0.594
	MIND diet * sex	0.091 (0.218)	0.676
**Model C**	MIND diet score (continuous)	-0.219 (0.112)	0.050
	APO-ε4 allele	1.578 (1.571)	0.315
	MIND diet * APO-ε4 allele	-0.197 (0.230)	0.392

Model A adjusted for age at death ((>= or <90 years), sex, education, calories, APO-ε4 allele, AD pathology, MIND diet, MIND diet*age at death.

Model B adjusted for age at death ((>= or <90 years), sex, education, calories, APO-ε4 allele, AD pathology, MIND diet, MIND diet*sex.

Model C adjusted for age at death ((>= or <90 years), sex, education, calories, APO-ε4 allele, AD pathology, MIND diet, MIND diet* APO-ε4 allele.

© 2025 Agarwal P et al. *JAMA Network Open*.

e.Table 5: Sensitivity analysis (after removing any FFQ done in last three years before death) for association of MIND diet with hippocampal sclerosis (HS). HS with Limbic-predominant age-related TDP-43 encephalopathy- neuropathological change (LATE-NC) and Hippocampal Neuronal loss severity.

	**OR (95% CI)**	**P for trend**
**Hippocampal Sclerosis**	N=630	
MIND diet score (continuous)	0.77 (0.63, 0.94)	-
MIND T1 (median score=6.0)	Ref	
MIND T2 (median score=7.0)	0.51 (0.27, 0.96)	
MIND T3 (median score=8.4)	0.51 (0.27, 0.94)	0.026
**Hippocampal Sclerosis with LATE-NC**	N=625	
MIND diet score (continuous)	0.80 (0.65, 0.98)	-
MIND T1 (median score=6.0)	Ref	
MIND T2 (median score=7.0)	0.56 (0.29, 1.09)	
MIND T3 (median score=8.4)	0.55 (0.29, 1.05)	0.066

© 2025 Agarwal P et al. *JAMA Network Open*.

**Hippocampal Neuronal Loss in Subsample ***	N=246	
MIND diet score (continuous)	0.80 (0.65, 1.00)	-
MIND T1 (median score=6.0)	Ref	
MIND T2 (median score=7.0)	0.52 (0.25, 1.07)	
MIND T3 (median score=8.4)	0.41 (0.28, 1.00)	0.017

All results for logistic models or *ordinal logistic model-models adjusted for age at death, sex, education, APO-ε4 allele, calories, AD pathology.

111 participants excluded from the model due to FFQ done in last three years before death.

e.Table 6. Spearman Correlation coefficient for MIND, Mediterranean and DASH diet scores in our analytical sample (N=809)

	MIND diet score	Mediterranean diet score	DASH diet score
MIND diet score	1	0.65 (p<0.001)	0.51 (p<0.001)
Mediterranean diet score		1	0.46 (p<0.001)

© 2025 Agarwal P et al. *JAMA Network Open*.

e.Table 7. Association of Mediterranean and DASH diet scores with hippocampal sclerosis (HS), HS with LATE, and hippocampal neuronal loss in our analytical sample

	Mediterranean diet score- OR (95% CI)		DASH diet score OR (95% CI)	
	**Model I (N=800)**	**Model II (n=739)**	**Model I (N=800)**	**Model II (N=739)**
**Hippocampal sclerosis**	0.93 (0.88, 0.98)	0.93 (0.87, 0.98)	0.84 (0.66, 1.05)	0.84 (0.66, 1.06)
**Hippocampal sclerosis with LATE-NC**	0.92 (0.87, 0.98)	0.92 (0.87, 0.98)	0.83 (0.65, 1.06)	0.83 (0.65, 1.08)
**Hippocampal neuronal loss**	0.95 (0.89,1.01)	0.96 (0.89, 1.03)	0.76 (0.57, 1.01)	0.84 (0.61, 1.15)

Both Mediterranean (range 0-55) and DASH diet (0-10) scores were assessed as continuous variables for main primary outcomes using logistic models adjusted for model I: age at death, sex, education, and calories and model II: model I +APO-ε4 allele, AD pathology and vascular pathology.

e.Figure 1: Marginal trajectory of the MIND diet score estimated in the years before death when considering a linear function of time or a non-linear function of time, MAP (n=809).

© 2025 Agarwal P et al. *JAMA Network Open*.

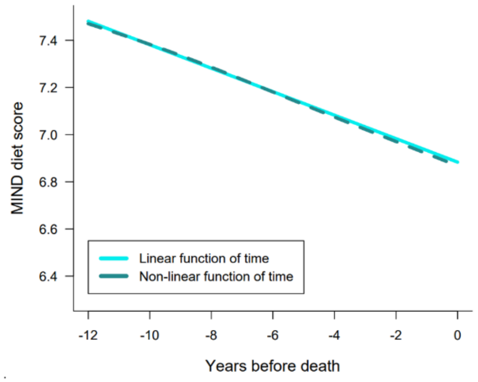

Years	≤-12	]-12;-10]	]-10;-8]	]-8;-6]	]-6;-4]	]-4;-2]	]-2;0]
Number of MIND observations	274	251	360	501	606	639	489

Linear trajectories were estimated over time (years before death) after controlling for sex, age at death, education, calories on both the intercept and the slope (i.e., interaction with time). Non-linear trajectories were estimated controlling for the same confounders but with the time approximated using flexible natural cubic splines with two inner knots located at the 33rd and 66th percentiles of the time distribution. Trajectories represent an average study participant profile (a woman aged 91 years-old at death with 15 years of education and daily intake of 1847 calories). When considering all years before death (range -15;0y), there is a decrease in MIND diet scores before death by -0.050 points per year; P<.0001.

e.Figure 2: Spline curves to test the non-linearity for the association of MIND diet score and risk of a) Hippocampal sclerosis (HS) b) HS with LATE-NC and c) hippocampal neuronal loss using natural cubic splines with two knots located at 33th and 66th percentile of the MIND diet score distribution.

© 2025 Agarwal P et al. *JAMA Network Open*.

a)

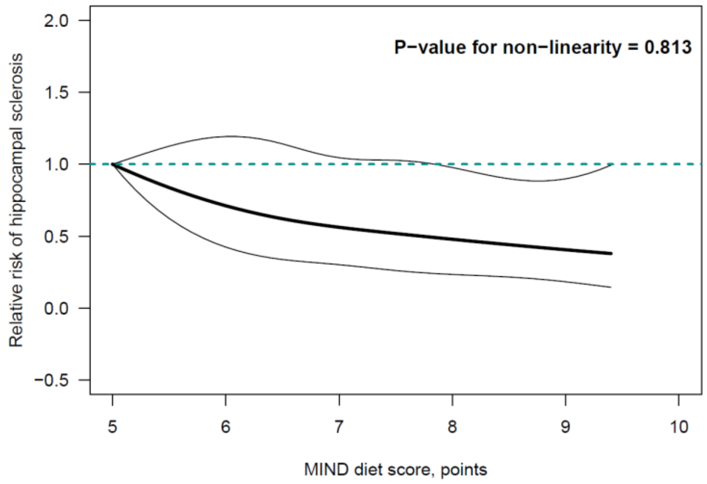

b)

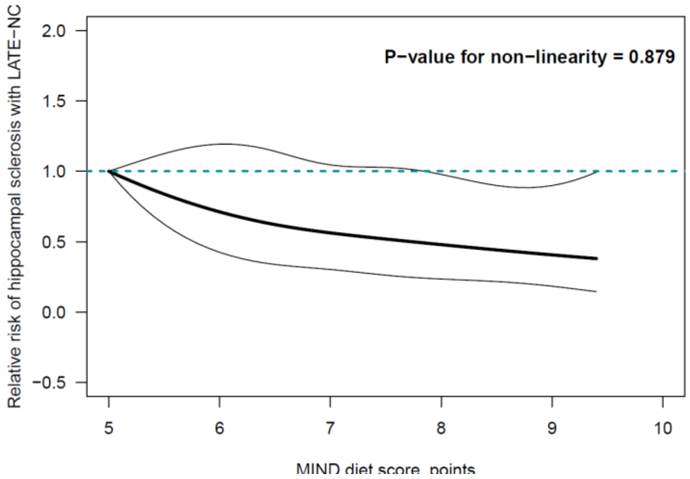

c)

All

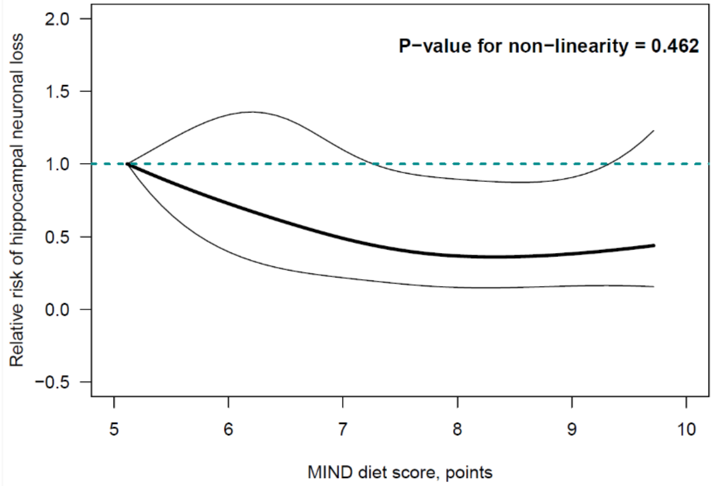

These models were adjusted for age at death (centered by the mean, 91 years), sex, education (centered by the mean, 15 years), calories (centered by the mean, 1847 kcal), APO-ε4 allele (present/ absent) and AD pathology (ADNC criteria yes/no). Dotted/ Lighter black lines represent 95% confidence intervals. the p-values for non-linearity were insignificant.

© 2025 Agarwal P et al. *JAMA Network Open*.
