# Supplementary material for: MIND Diet and Hippocampal Sclerosis Among Community-Based Older Adults
Source: JAMA Netw Open. 2025 Aug 7;8(8):e2526089. doi: 10.1001/jamanetworkopen.2025.26089 (PMC12332637; doi:10.1001/jamanetworkopen.2025.26089)
Supplement: Supplement 2. — Data Sharing Statement [file jamanetwopen-e2526089-s002.pdf]

Data Sharing Statement

Agarwal. MIND Diet and Hippocampal Sclerosis Among Community-Based Older Adults. *JAMA Netw Open*. Published August 07, 2025. doi:10.1001/jamanetworkopen.2025.26089

Data
Data available: Yes
Data types: Deidentified participant data
How to access data: https://www.radc.rush.edu/
When available: With publication

Supporting Documents
Document types: None

Additional Information
Who can access the data: researchers whose proposed use of the data has been approved
Types of analyses: researchers whose proposed use of the data has been approved
Mechanisms of data availability: after approval of a proposal, and with a signed data access agreement
Any additional restrictions: -
